# Heme oxygenase-1 deficiency presenting with interstitial lung disease and hemophagocytic flares

**DOI:** 10.1186/s12969-020-00474-1

**Published:** 2020-10-16

**Authors:** Alice S. Chau, Bonnie L. Cole, Jason S. Debley, Kabita Nanda, Aaron B. I. Rosen, Michael J. Bamshad, Deborah A. Nickerson, Troy R. Torgerson, Eric J. Allenspach

**Affiliations:** 1 Division of Allergy & Infectious Disease, Department of Medicine, University of Washington, Seattle, Washington USA; 2 Center for Immunity and Immunotherapies, Seattle Children’s Research Institute, Jack MacDonald Building – 6th floor, 1900 9th Avenue, Seattle, Washington 98101 USA; 3 Department of Pathology and Laboratory Medicine, University of Washington, Seattle, Washington USA; 4 Brotman Baty Institute for Precision Medicine, Seattle, Washington USA; 5 Department of Pediatrics, University of Washington, Seattle, Washington USA; 6 Genome Sciences, University of Washington, Seattle, Washington USA; 7 Experimental Immunology, Allen Institute, Seattle, Washington USA

**Keywords:** HMOX1, Heme oxygenase-1, HO-1, NSIP, Systemic juvenile idiopathic arthritis, Macrophage activation syndrome, Asplenia, Hemophagocytosis lymphohistiocytosis, Vasculitis

## Abstract

**Background:**

Heme oxygenase-1 (HMOX1) catalyzes the metabolism of heme into carbon monoxide, ferrous iron, and biliverdin. Through biliverdin reductase, biliverdin becomes bilirubin. *HMOX1*-deficiency is a rare autosomal recessive disorder with hallmark features of direct antibody negative hemolytic anemia with normal bilirubin, hyperinflammation and features similar to macrophage activation syndrome. Clinical findings have included asplenia, nephritis, hepatitis, and vasculitis. Pulmonary features and evaluation of the immune response have been limited.

**Case presentation:**

We present a young boy who presented with chronic respiratory failure due to nonspecific interstitial pneumonia following a chronic history of infection-triggered recurrent hyperinflammatory flares. Episodes included hemolysis without hyperbilirubinemia, immunodeficiency, hepatomegaly with mild transaminitis, asplenia, leukocytosis, thrombocytosis, joint pain and features of macrophage activation with negative autoimmune serologies. Lung biopsy revealed cholesterol granulomas. He was found post-mortem by whole exome sequencing to have a compound heterozygous paternal frame shift a paternal frame shift HMOX1(NM_002133.3):c.262_268delGCCCTGGinsCC (p.Ala88Profs*51) and maternal splice donor *HMOX1* (c.636 + 2 T > A) consistent with HMOX1 deficiency. Western blot analysis confirmed lack of HMOX1 protein upon oxidant stimulation of the patient cells.

**Conclusions:**

Here, we describe a phenotype expansion for HMOX1-deficiency to include not only asplenia and hepatomegaly, but also interstitial lung disease with cholesterol granulomas and inflammatory flares with hemophagocytosis present in the bone marrow.

## Background

Heme oxygenases are rate-limiting enzymes that catalyze the degradation of heme to carbon monoxide (CO), ferrous iron, and biliverdin, which then becomes bilirubin via the action of biliverdin reductase. Two isoforms exist, heme oxygenase-1 (HMOX1) and heme oxygenase-2 (HMOX2), with CO, biliverdin, and bilirubin implicated in important cellular processes, such as inflammation, cell proliferation, apoptosis, and antioxidant defense. HMOX1 is distributed in the liver, spleen, and endothelium with rapid induction in the presence of stressors, while HMOX2 expression is widespread and cannot be induced. HMOX1 was first discovered in the 1968 [1] but the first case of HMOX1 deficiency was not described until 1999 [2].

HMOX1 deficiency is an extremely rare autosomal recessive disorder with a small number of cases reported to date [2–8] (Supplemental Table 1). The rarity may derive from the role of fetal HMOX1 in placental health [9, 10]. Clinical presentation is complex and diverse, including direct antibody negative hemolytic anemia, low bilirubin, and hyperinflammation [3]. HMOX1 is induced in the liver, spleen and endothelium after oxidative stressors and hypoxia [3]. One reported case appeared to mimic vasculitis and another was thought to have hemophagocytic lymphohistiocytosis (HLH). Diagnosis of HMOX1 deficiency lies within clinical findings and laboratory studies with genetic testing of *HMOX1* required for confirmation.

Here, we describe a boy born to nonconsanguineous parents who presented with early onset asplenia, recurrent infections, and associated flares with bone marrow histiocyte activation with worsening interstitial lung disease and joint pain.

## Case presentation

A 10-year-old boy was admitted for diagnostic lung biopsy in the setting of progressive chronic hypoxic respiratory failure and recurrent hyperinflammatory episodes. He was born at 7 pounds 3 oz at estimated gestational age of 36 weeks via normal spontaneous vaginal delivery to a mother with a history of placental clots with a still birth at term. He was hospitalized at 4 months of age for respiratory syncytial virus (RSV) for 7 days, at 1 year old for hypospadias repair, and then again at age 3 years 8 months for what was thought to be mononucleosis due to positive Epstein-Barr virus (EBV) positive immunoglobulin M (IgM). During the latter episode, he was severely fatigued and had persistent fevers to 40 °C. Additionally, he had another RSV infection at 3 years and 4 months of age. He demonstrated mild gross motor developmental delay as he did not crawl and did not walk until 19 months of age. He received all regularly scheduled vaccines until 3 years of age, but subsequently stopped regular vaccination.

At approximately 4 years of age, he presented with a one-month history of fatigue, intermittent fevers and dark urine. His fevers were daily reaching 40 °C with periods of defervescence. He then developed a cough with hypoxemia to 89% on room air and was admitted for viral bronchiolitis. Physical exam was notable for mild prognathism, slight frontal prominence, low-set and posteriorly rotated ears, mild pectus excavatum, bilateral undescended testes, and long fingers and toes with overlapping second and fourth toes over the third toes bilaterally were noted. His elbows and knees were hyperextensible and demonstrated moderate pes planus and out-toeing.

During hospitalization, hepatomegaly was found along with mild transaminitis (AST 301 U/L, ALT 74 U/L), direct antiglobulin test negative hemolytic anemia (hematocrit 24.7%) and hemoglobinuria without microscopic red blood cells. Abdominal CT scan revealed a small poorly perfused spleen which correlated well with the Howell-Jolly bodies and schistocytes on peripheral smear. Bilirubin was normal but lactate dehydrogenase (LDH) was dramatically elevated at 19,706 U/L. Normal renal function was present with creatinine 0.1 mg/d without evidence of proteinuria or myoglobinuria. Creatine kinase values were normal at 202 IU/L. Systemic inflammation was present with leukocytosis (peak 53.8 K/mm^3^), thrombocytosis (peak 914 k/mm^3^), elevated erythrocyte sedimentation rate (ESR, 87 mm/hr), hyperferritinemia to 1980 ng/mL, but blood cultures and respiratory viral PCR panel was negative.

He had a liver biopsy that demonstrated mild sinusoidal fibrosis, mild microvesicular steatosis, and rare apoptotic hepatocytes, but ultimately was non-diagnostic. Work up for hypercoagulability, serum muscle enzymes and amino acid and organic acids from the urine and plasma were all normal. Serologies for antiphospholipid antibody syndrome, antineutrophil cytoplasmic antibodies, anti-nuclear antibody, anti RNP, and anti-SSA/SSB were all negative. Autoimmune hepatitis work-up yielded negative liver kidney microsomal and smooth muscle antibodies. Respiratory symptoms slowly resolved and hematologic findings improved, thus representing a flare that recurred regularly over the next 6 years ranging from 4 to 17 weeks duration mainly treated with steroids.

During his next flare, the patient had anemia, leukocytosis, and thrombocytosis along with abdominal pain, hepatomegaly, and fevers. Further imaging with CTA abdomen demonstrated absent splenic veins and multiple collaterals to a small left kidney, implying that patient’s spleen had infarcted. A bone marrow biopsy demonstrated extensive histiocyte activation with phagocytosis of nucleated red blood cell precursors. There was normal cellularity but decreased trilineage hematopoiesis and increased megakaryocytes; no malignant cells were present. This flare was associated with HHV-7 viremia.

**Fig. 1:**
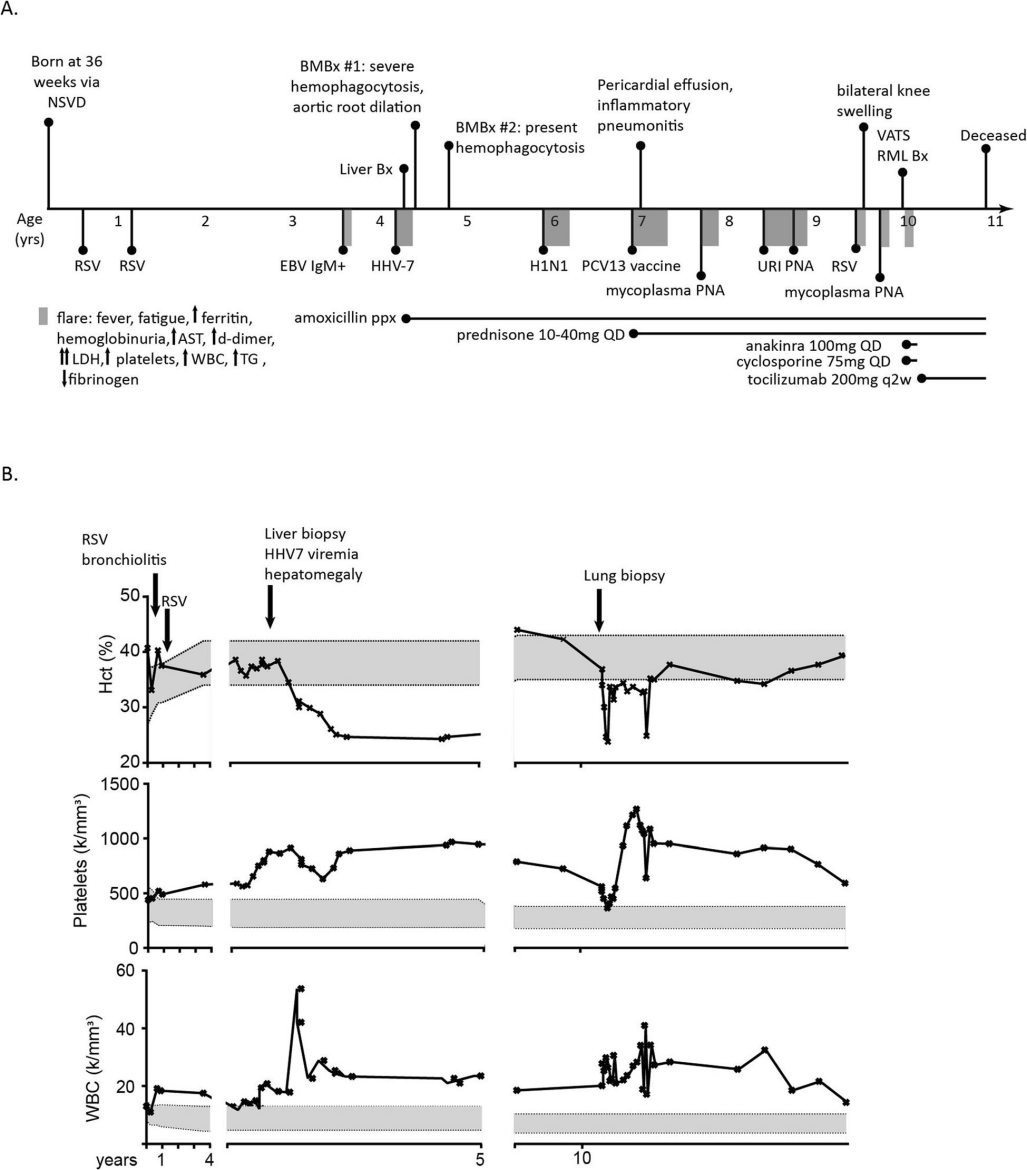
Hematologic values at baseline and during flares. **a** Clinical timeline with major events (*above*) and infections (*below*). The flares duration is indicated in shaded box. **b** Trends of patient’s laboratory values for white blood count (WBC), platelets (Plts), and hematocrit (Hct) over the clinical course. Age-specific reference values are noted in grey shaded in between the upper and lower limits of normal (dotted lines). The timeline shown in years is broken into early childhood (0-4 years) and then two flare episodes with numerous values to compare (4.5–5 years and 9.5-10 years). Known events immediately preceding flares are indicated (arrows)

He was readmitted to the hospital multiple times for similar febrile episodes found to be triggered by viral and bacterial infections as well as Prevnar vaccination **(**Fig. 1**)**. He had a prolonged four-month long flare following H1N1 infection complicated by pneumonia with pleural effusion. He received the Prevnar 13 vaccination and developed another hyperinflammatory episode lasting 4 months complicated by steroid responsive pericardial effusion and presumed inflammatory pneumonitis. He soon became oral corticosteroid-dependent as weaning resulted in hemolysis and dark urine. By the age of 8, the flares were characterized less by persistent febrile episodes but more by shortness of breath, chest discomfort and intermittent desaturations. His growth curve had started to plateau at age 4 despite being at the 50th percentile until the age of 3; he was less than the 10th percentile for weight and 20th percentile for height. He also began experiencing hip pain with unequal leg lengths, difficulty running, and decreased stamina. Bilateral knee arthritis was clinically noted accompanying myalgias and arthralgias with morning stiffness, although subsequent knee x-rays showed no erosions. Mild proteinuria developed as well. He was steroid responsive and therefore treated with oral prednisone 10 mg twice daily. Steroid sparing therapies, such as methotrexate and azathioprine, were briefly introduced but discontinued because no benefit was observed.

Due to persistent and progressive respiratory symptoms exacerbated by an infection with RSV and mycoplasma, he was hospitalized at Seattle Children’s Hospital for further evaluation. Spirometry testing demonstrated a severely restrictive pulmonary pattern with a forced vital capacity (FVC) of 0.41 L (20%), forced expiratory volume in 1 s (FEV1) of 0.41 (22%), and FEV1/FVC 99%. He underwent a right thoracoscopic lung biopsy, which demonstrated extensive fibrotic nonspecific interstitial pneumonia (NSIP), patchy pleural fibrosis, and scattered cholesterol granulomas.

Following the procedure, he developed a right hemothorax and pneumothorax with respiratory distress and supplemental oxygen, requiring Pediatric Intensive Care Unit (PICU) admission. He had substantial fibrotic intrathoracic tissue and his pulmonary function continued to deteriorate, requiring consistent use of nasal cannula and increased use of BiPAP. To treat his inflammatory state, corticosteroid dose was increased and gradually weaned while anti-IL-1R therapy (anakinra), was trialed for 10 days, overlapping with cyclosporine, and then switched to anti-IL-6 therapy (tocilizumab) with minimal benefit. He expired just prior to his eleventh birthday due to respiratory failure.

### Patient laboratory, histopathology, and radiologic evaluation

During episodes, his baseline leukocytosis increased from about 20 K/mm^3^ to exceed 40 K/mm^3^. Hyposplenia, initially noted at age 4, was confirmed on serial abdominal imaging, contributed to baseline thrombocytosis, but platelet counts exceeded 1 million frequently during flares, requiring aspirin for coagulation prophylaxis. At baseline, he had mild anemia with hematocrit of high 30%/low 40s%. However, during flares, his hematocrit would nadir below 30%. LDH was elevated at baseline and episodically reached 28,000 U/L with uniformly elevated isoenzymes. His transaminitis largely remained within the mild range with corresponding mild elevation of GGT and INR **(**Table 1**)**. Alpha-1 antitrypsin was normal at 245 mg/dL as was alpha fetoprotein (0.9 ng/mL). Metabolic etiologies were ruled out with plasma and urine amino acid levels as well as urine organic acid levels. At no point did he have gastrointestinal or central nervous system involvement.

**Table 1:** Laboratory studies. Patient laboratory values are displayed for the patient for the ranges from hospitalizations at our institution with the normal value ranges for each indicated test listed

Test	Normal Values	Patient’s Values
**Biochemical**		
ALT	5–41 IU/L	165–615
AST	6–40 IU/L	44–157
GGT	15–85 IU/L	30–405
Total bilirubin	0.0–1.1 mg/dL	0.2
INR	< 1.0	1.2–1.6
Fibrinogen	230–450 mg/dL	57–493
D-dimer	≤0.5 mg FEU/mL	> 20
Ceruloplasmin	29–56 mg/dL	48
Liver copper	10–35 μg/g dry weight	43
Plasma copper	56–191 mcg/dL	191
Triglycerides	60–135 mg/dL	95–503
LDL	< 110 mg/dL	324
HDL	> 39 mg/dL	58
Total LDH	145–345 U/L	5490–28,019
LDH 1 (%)	17.5–28.3% (I, Heart)	7.5–10
LDH 2 (%)	30.4–36.4% (II)	17.6–21
LDH 3 (%)	19.2–24.8% (III)	26.9
LDH 4 (%)	9.6–15.6% (IV)	23.7
LDH 5 (%)	5.5–12.7% (V, Liver)	24.3
Ferritin	10–300 ng/mL	555–4264
sIL-2R	45–1105 U/mL	145
**Immunological**		
IgG	608–1572 mg/dL	1050–1140
IgA	52–242 mg/dL	261
IgM	45–236 mg/dL	89–108
IgE	0.98–570.6 mg/dL	105
IgD	≤10 mg/dL	3
PPSV23 (PCV13)	≥8/21 (≥5/12)	8/21 (5/12)
Tetanus	≥0.01 IU/mL	0.43
C3	83–203 mg/dL	186
C4	16–52 mg/dL	24
CH50	> 32 unit/mL	69
CD3	1200-2600/mm^3^	6086; 1465
CD4	650–1500/mm^3^	3793; 786
CD8	370–1100/mm^3^	2117; 661
CD4:CD8	> 2:1	1:8:1; 1.2:1
CD16^+^CD56^+^	120–480/mm^3^	882; 89
CD19	270–860/mm^3^	1588; 232
PHA	> 30%	24.70%
anti-CD3	> 30%	21.50%
NK function		
50:1	> 20	11
25:1	> 10	9
12.5:1	> 5	5
6.25:1	> 1	5
Lytic units	> 3.1	2.5

Abbreviations: *ALT* Alanine aminotransferase, *AST* Aspartate aminotransferase, *CH50* Total hemolytic complement activity, *GGT* γ-glutamyl transferase, *HDL* High density lipoprotein, *IgA* Immunoglobulin A, *IgD* Immunoglobulin D, *IgG* Immunoglobulin G, *IgM* Immunoglobulin M, *INR* International normalized ratio, *LDH* Lactate dehydrogenase, *LDL* Low density lipoprotein, *NK* Natural killer cell, *PHA* Phytohemagglutinin, *PPSV23* Pneumococcal vaccine polyvalent, *sIL-2R* Soluble interleukin-2 receptor

**Fig. 2:**
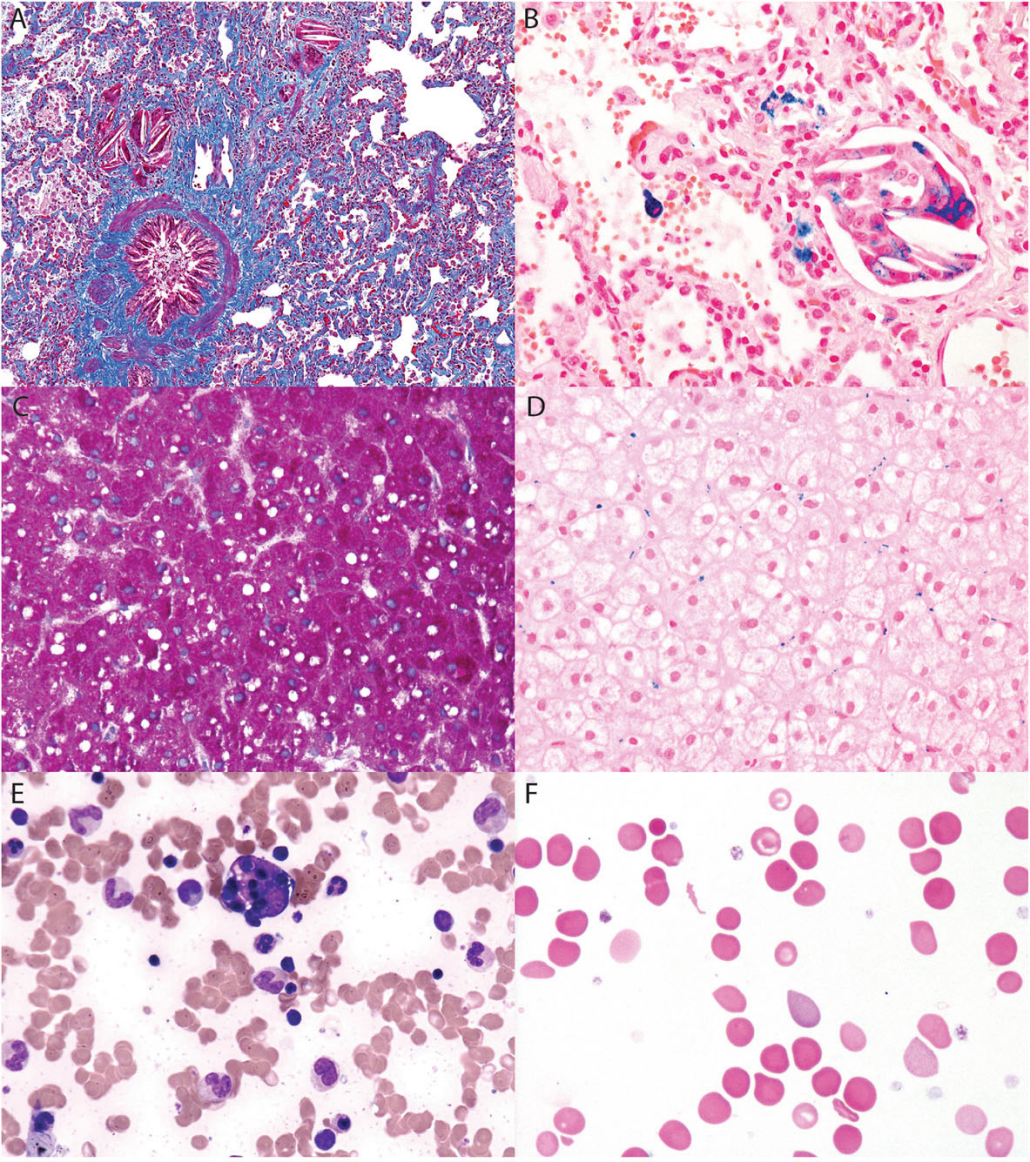
Histopathology demonstrating unique features of HMOX1 deficiency. **a** Trichrome stained sections from lung biopsy tissue demonstrate extensive alveolar septal fibrosis and scattered granulomas. **b** Iron staining of lung tissue highlights hemosiderin laden macrophages (blue granules) associated with cholesterol granulomas. **c** Trichrome stained liver biopsy with mild sinusoidsal fibrosis and microvesicular steatosis and (**d**) iron stained liver biopsy with increased iron (blue granules) in Kupffer cells (blue). **e** Wright stained bone marrow aspirate demonstrating hemophagocytosis. **f** Peripheral blood smear demonstrating anisocytosis, schistocytes, elliptocytes, and a Howell-Jolly body

During two separate hospitalizations for flares, the diagnosis of hemophagocytic lymphohistiocytosis (HLH) and macrophage activation syndrome (MAS) were both considered based upon his laboratory features. Overall, two bone marrow biopsies were performed approximately 1 year apart, and both demonstrated normal cellularity and markedly increased hemophagocytosis **(**Fig. 2**)**. Natural killer (NK) cell function was assessed and was decreased **(**Table 1**)**. CD107a could not be assessed due to insufficient NK cells. Soluble IL-2 receptor (sIL-2R, also known as soluble CD25) was normal and never elevated. Genetic testing for periodic fever syndromes and familial HLH were performed, but no pathogenic variants in known genes were identified. Comparative genomic hybridization (CGH) revealed no structural variants, and he had a normal male karyotype. Treatment escalation was not required as the symptoms gradually waned with continued prednisone.

Given his recurrent infections, an immune evaluation was performed revealing abnormal T cell proliferation to stimulation with both phytohemagglutinin (PHA) and anti-CD3 **(**Table 1**)**. He had increased naïve CD45RA^+^CD27^+^CCR7^+^ population (65% of cells), few effector memory T cells, and likewise immature CD8^+^ population with > 65% of the cells naïve. He had normal quantitative immunoglobulin levels and robust vaccine responses, but B cell immunophenotyping was notable for absent immature and transitional B cells with reduced CD27^+^ memory B at 6% (normal > 8%). Class switched and BAFF receptor populations were normal. Further T cell analysis was not performed.

### Genetic analysis

**Fig. 3:**
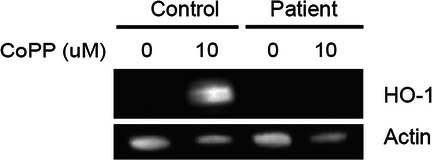
Patient cells lacked HMOX protein expression. Representative western blot analysis of HMOX1 following induction with cobalt protoporphyrin (CoPP) for 24 h of patient’s peripheral blood mononuclear cells compared to control cells. Patient is demonstrated to lack expression of HMOX1

Whole exome sequencing of patient, mother, father, and brother were performed revealing a compound heterozygous paternal frame shift HMOX1(NM_002133.3):c.262_268delGCCCTGGinsCC (p.Ala88Profs*51) and maternal splice donor *HMOX1* (c.636 + 2 T > A) consistent with HMOX1 deficiency. Western blot analysis subsequently confirmed that cells treated with a known inducer of HMOX1, Cobalt protoporphyrin (CoPP), resulted in no protein was expressed **(**Fig. 3**)**, confirming HMOX1 deficiency.

## Discussion

The boy reported herein is a case of HMOX1 deficiency notable for the presence of chronic pulmonary disease and inflammatory flares with notable hemophagocytosis. He was found on thorascopic lung biopsy to have extensive interstitial fibrosis, consistent with the fibrotic nonspecific interstitial pneumonia (NSIP) pattern, in addition to cholesterol granulomas. NSIP is a diffuse lung disease that may have a cellular, fibrotic, or mixed pattern. It is the most common of the diffuse lung diseases in the pediatric population often associated with a systemic disease. The majority of diffuse lung diseases are attributed to connective tissue disorders, such as systemic lupus erythematous, polymyositis/dermatomyositis, systemic sclerosis, mixed connective tissue disease, and systemic juvenile idiopathic arthritis (sJIA) [11, 12]. Surfactant disorders also account for many interstitial lung disease cases in both pediatrics and adults previously thought to be idiopathic [13] .

Cholesterol granulomas are also rare, especially in children. Pulmonary interstitial and intra-alveolar cholesterol granulomas (PICG) are formed when degenerating macrophages release cholesterol esters in the interstitium and with organization form granulomas. The cholesterol appears as acicular crystals on light microscopy **(**Fig. 2**)**. PICG typically appears in the setting of lipoid pneumonia with or without pulmonary alveolar proteinosis [14]. Exogenous lipoid pneumonia results from inhalation or aspiration of mineral, plant or animal-based oils, and/or ascending aspiration of such oils in the setting of gastroesophageal reflux [15, 16] . In this case, there was no history suggestive of exogenous oil aspiration or gastroesophageal reflux. However, PICGs due to endogenous etiologies without lipoid pneumonia are very rare and has been reported in pulmonary hypertension [17, 18] or in the setting of sJIA [19].

Our patient developed severe NSIP, likely due to oxidant-induced injury [20], which has not been reported in other patients with HMOX1 deficiency. In a post-mortem analysis of one patient, there were microthrombi in the arterioles and capillaries of the lungs with focal alveolitis, but no chronic lung changes. In another case, there was diffuse alveolar hemorrhage reported with suspicion of small vessel vasculitis and yet another case reported HMOX1 deficiency as a mimic of childhood vasculitis outside the lungs [6] [5]. Although oxidant-induced lung injury has been discussed in murine models of HMOX1 deficiency, previously reported patients did not develop chronic pulmonary complications prior to their death (Supplemental Table 1). The pulmonary features in our case showed progressive fibrosis and cholesterol granulomas that may be related to the macrophage activation as similar histology has been reported in sJIA.

The lung biopsy of our patient and presence of hemophagocytes in the bone marrow were consistent with sJIA [21], a diagnosis of exclusion, but our patient has only one episode of clinically documented arthritis. Hemophagocytosis is frequently associated with macrophage activation syndrome (MAS), a rare and potentially fatal complication of sJIA [22]. Our patient met the 2016 classification criteria for MAS based upon febrile patient suspected of sJIA with elevated ferritin, AST, and TG and depressed fibrinogen [23]. Hemophagocytosis can also be observed acutely in infection and malignancy, although the chronicity of his condition and extensive malignancy work-up made these conditions less likely. Lastly, rare inborn errors of metabolism have also been rarely associated with hemophagocytosis, including lysosomal storage disorders such as Gaucher disease [24], organic acidemia [25], or Wolman disease [26]. As such, screening and genetic tests for lysosomal enzyme function, fibroblast cultures, and urine mucopolysaccharides and oligosaccharides were performed in our patient but were normal.

Several cases of HMOX1 deficiency have been reported as a mimic of HLH and treated as such given more acute courses and meeting HLH criteria [6, 8, 27]. One case demonstrated absent NK cell function in the setting of persistent fevers, hypertriglyceridemia, hyperferritinemia, and elevated sIL-2R. Our patient had a chronic course with later flares lacking fever. During flares, our patient had features of HLH including hepatomegaly, hemophagocytosis in the bone marrow, absent NK cell functional activity, and hyperferritinemia. Genetic HLH panel testing was sent but no pathogenic variants were identified. Acute presentations of HMOX1 deficiency share significant features with both MAS and HLH.

Immune evaluations were not performed in prior patients with HMOX1 deficiency. Asplenia is commonly reported as a bacterial infection risk, but our patient had more notable viral infections. Although our patient demonstrated mild impairment in mitogen and anti-CD3 stimulation, the clinical assay cannot distinguish cell death from poor proliferation. No overt quantitative or qualitative humoral defects were identified in our subject, but both B and T lymphocytes was skewed away from immature to mature immunophenotype, which can be seen in chronic inflammatory disease. The role in T cell and NK cell function will be important to clarify in HMOX1 deficiency.

HMOX1 deficiency results in overt heme concentrations, low bilirubin, and marked oxidative stress with varied phenotype rooted in hemolytic anemia, low bilirubin, and hyperinflammation. TLR9 in mice has been found to induce HMOX1 expression in bone marrow dendritic cells, which in turn regulates macrophage production of IL-10 that is highly involved in MAS when dysregulated [28]. Furthermore, the defect in HMOX1 putatively impairs phagocytosis with a murine study demonstrating subablative bone marrow transplantation of HMOX1 deficient mice reverses disease due to repopulation of wild type macrophages [29]. Therefore, while speculative, myeloablative bone marrow transplantation may be a treatment option for these children with HMOX1 deficiency.

## Conclusions

Here we report a young man with HMOX1 deficiency that had recurrent autoinflammatory episodes marked by fever, hemolysis and hyperferritinemia with pathologic features similar to MAS and HLH.

Our case highlights that HMOX1 deficiency can also have marked lung disease resulting in early mortality.

## Methods

### Subjects

Subjects were consented into the Genetic Basis of Immunodeficiency Diseases Biorepository at the Seattle Children’s Hospital (IRB #11738) and consented for the University of Washington Repository for Mendelian Disorders for genetic studies approved by the University of Washington Institute Review Board all in compliance with database of Genotypes and Phenotypes (dbGaP).

### Whole exome sequencing

Whole exome sequencing was performed in collaboration with University of Washington Center for Mendelian Genomics (UWCMG) on our quad family with one affected proband, unaffected brother, father, and mother. Sanger sequencing also confirmed the variants.

### Western blot

Primary peripheral blood mononuclear cells were stimulated with 10 μM Cobaltic Protoporphyrin IX Chloride (Santa Cruz Biotechnologies #sc-294,098, Santa Cruz, CA) for 24 h. RIPA lysates (Thermo Fisher #89900) were run on NuPAGE 4–12% gradient Bis-Tris Protein gels (Thermo #NP0322) and transferred to nitrocellulose blocked using Odyssey Blocking Buffer (LiCor #927–40,000) and stained using anti-Human/Mouse HO-1/HMOX1 (R&D #MAB3776)[Monoclonal Rat IgG_2B_ Clone # 412811] at 1:1000 dilution and detected using Odyssey anti-rat IgG (H + L) IRDye 800CW secondary reagent (1:15,000).

## Supplementary information

**Additional file 1: Table S1.** Summary of previously published *HMOX1* deficiency cases.

## Data Availability

Not applicable.

## Abbreviations

CGH: Comparative genomic hybridization (CGH)
CH50: Total hemolytic complement activity
CoPP: Cobalt protoporphyrin
EBV: Epstein Barr virus
ESR: Erythrocyte sedimentation rate
FEV1: Forced expiratory volume in 1 s
FVC: Forced vital capacity
GGT: γ-glutamyl transferase
HDL: High density lipoprotein
HHV7: Human Herpesvirus 7
HLH: Hemophagocytic lymphohistiocytosis
HMOX1: Heme oxgenase-1
IgA: Immunoglobulin A
IgD: Immunoglobulin D
IgG: Immunoglobulin G
IgM: Immunoglobulin M
INR: International normalized ratio
LDH: Lactate dehydrogenase
LDL: Low density lipoprotein
MAS: Macrophage activation syndrome
NK: Natural killer
NSIP: Nonspecific interstitial pneumonia
PHA: Phytohemagglutinin
PICG: Pulmonary interstitial and intra-alveolar cholesterol granulomas
PNA: Pneumonia
RSV: Respiratory syncytial virus
RSV: Respiratory syncytial virus
sIL-2R: Soluble interleukin-2 receptor
sJIA: Systemic juvenile idiopathic arthritis

## References

[1] Tenhunen R, Marver HS, Schmid R (1968). The enzymatic conversion of heme to bilirubin by microsomal heme oxygenase. Proc Natl Acad Sci U S A.

[2] Yachie A, Niida Y, Wada T, Igarashi N, Kaneda H, Toma T (1999). Oxidative stress causes enhanced endothelial cell injury in human heme oxygenase-1 deficiency. J Clin Invest.

[3] Kawashima A, Oda Y, Yachie A, Koizumi S, Nakanishi I (2002). Heme oxygenase-1 deficiency: the first autopsy case. Hum Pathol.

[4] Radhakrishnan N, Yadav SP, Sachdeva A, Pruthi PK, Sawhney S, Piplani T (2011). Human heme oxygenase-1 deficiency presenting with hemolysis, nephritis, and asplenia. J Pediatr Hematol Oncol.

[5] Radhakrishnan N, Yadav SP, Sachdeva A, Wada T, Yachie A (2011). An interesting tetrad of asplenia, inflammation, hemolysis, and nephritis. Pediatr Hematol Oncol.

[6] Gupta A, Akihiro Y, Saxena AK, Bhattacharya A, Singh S (2016). Haem oxygenase-1 deficiency: a mimicker of childhood vasculitis. Scand J Rheumatol.

[7] Greil J, Verga-Falzacappa MV, Echner NE, Behnisch W, Bandapalli OR, Pechanska P (2016). Mutating heme oxygenase-1 into a peroxidase causes a defect in bilirubin synthesis associated with microcytic anemia and severe hyperinflammation. Haematologica..

[8] Tahghighi F, Parvaneh N, Ziaee V (2019). Post-mortem diagnosis of Heme Oxygenase-1 deficiency by whole exome sequencing in an Iranian child. Int J Mol Cell Med.

[9] Ozen M, Zhao H, Lewis DB, Wong RJ, Stevenson DK (2015). Heme oxygenase and the immune system in normal and pathological pregnancies. Front Pharmacol.

[10] Tsur A, Kalish F, Burgess J, Nayak NR, Zhao H, Casey KM (2019). Pravastatin improves fetal survival in mice with a partial deficiency of heme oxygenase-1. Placenta..

[11] Fan LL, Dishop MK, Galambos C, Askin FB, White FV, Langston C (2015). Diffuse lung disease in biopsied children 2 to 18 years of age. Application of the chILD classification scheme. Ann Am Thorac Soc.

[12] Cottin V, Hirani NA, Hotchkin DL, Nambiar AM, Ogura T, Otaola M (2018). Presentation, diagnosis and clinical course of the spectrum of progressive-fibrosing interstitial lung diseases. Eur Respir Rev.

[13] Nathan N, Borensztajn K, Clement A (2018). Genetic causes and clinical management of pediatric interstitial lung diseases. Curr Opin Pulm Med.

[14] Fisher M, Roggli V, Merten D, Mulvihill D, Spock A (1992). Coexisting endogenous lipoid pneumonia, cholesterol granulomas, and pulmonary alveolar proteinosis in a pediatric population: a clinical, radiographic, and pathologic correlation. Pediatr Pathol.

[15] Marangu D, Gray D, Vanker A, Zampoli M. Exogenous lipoid pneumonia in children: a systematic review. Paediatr Respir Rev. 2020;33:45–51. 10.1016/j.prrv.2019.01.001 PMC7106224 30962152

[16] Hu X, Lee JS, Pianosi PT, Ryu JH (2015). Aspiration-related pulmonary syndromes. Chest..

[17] Glancy DL, Frazier PD, Roberts WC (1968). Pulmonary parenchymal cholesterol-ester granulomas in patients with pulmonary hypertension. Am J Med.

[18] Fischer EG, Marek JM, Morris A, Nashelsky MB (2000). Cholesterol granulomas of the lungs associated with microangiopathic hemolytic anemia and thrombocytopenia in pulmonary hypertension. Arch Pathol Lab Med.

[19] Schultz R, Mattila J, Gappa M, Verronen P (2001). Development of progressive pulmonary interstitial and intra-alveolar cholesterol granulomas (PICG) associated with therapy-resistant chronic systemic juvenile arthritis (CJA). Pediatr Pulmonol.

[20] Choi AM, Alam J (1996). Heme oxygenase-1: function, regulation, and implication of a novel stress-inducible protein in oxidant-induced lung injury. Am J Respir Cell Mol Biol.

[21] Saper VE, Chen G, Deutsch GH, Guillerman RP, Birgmeier J, Jagadeesh K (2019). Emergent high fatality lung disease in systemic juvenile idiopathic arthritis. Ann Rheum Dis.

[22] Minoia F, Davi S, Horne A, Demirkaya E, Bovis F, Li C (2014). Clinical features, treatment, and outcome of macrophage activation syndrome complicating systemic juvenile idiopathic arthritis: a multinational, multicenter study of 362 patients. Arthritis Rheumatol.

[23] Ravelli A, Minoia F, Davi S, Horne A, Bovis F, Pistorio A (2016). Ann Rheum Dis.

[24] Sharpe LR, Ancliff P, Amrolia P, Gilmour KC, Vellodi A (2009). Type II Gaucher disease manifesting as haemophagocytic lymphohistiocytosis. J Inherit Metab Dis.

[25] Gokce M, Unal O, Hismi B, Gumruk F, Coskun T, Balta G (2012). Secondary hemophagocytosis in 3 patients with organic acidemia involving propionate metabolism. Pediatr Hematol Oncol.

[26] Taurisano R, Maiorana A, De Benedetti F, Dionisi-Vici C, Boldrini R, Deodato F (2014). Wolman disease associated with hemophagocytic lymphohistiocytosis: attempts for an explanation. Eur J Pediatr.

[27] Henter JI, Horne A, Arico M, Egeler RM, Filipovich AH, Imashuku S (2007). HLH-2004: diagnostic and therapeutic guidelines for hemophagocytic lymphohistiocytosis. Pediatr Blood Cancer.

[28] Biswas C, Burn T, Chu N, Behrens E. Monomethyl fumarate as a novel therapy for macrophage activation syndrome: mechanism of action in an animal model [abstract]. Arthritis Rheumatol. 2019;71(suppl 10). https://acrabstracts.org/abstract/monomethyl-fumarate-as-a-novel-therapy-for-macrophage-activation-syndrome-mechanism-of-action-in-an-animal-model/.

[29] Kovtunovych G, Ghosh MC, Ollivierre W, Weitzel RP, Eckhaus MA, Tisdale JF (2014). Wild-type macrophages reverse disease in heme oxygenase 1-deficient mice. Blood..

