# Correction to: Heme oxygenase-1 deficiency presenting with interstitial lung disease and hemophagocytic flares

**DOI:** 10.1186/s12969-021-00661-8

**Published:** 2022-03-14

**Authors:** Alice S. Chau, Bonnie L. Cole, Jason S. Debley, Kabita Nanda, Aaron B. I. Rosen, Michael J. Bamshad, Deborah A. Nickerson, Troy R. Torgerson, Eric J. Allenspach

**Affiliations:** 1 Division of Allergy & Infectious Disease, Department of Medicine, University of Washington, Seattle, Washington USA; 2 Center for Immunity and Immunotherapies, Seattle Children’s Research Institute, Jack MacDonald Building – 6th floor, 1900 9th Avenue, Seattle, Washington 98101 USA; 3 Department of Pathology and Laboratory Medicine, University of Washington, Seattle, Washington USA; 4 Brotman Baty Institute for Precision Medicine, Seattle, Washington USA; 5 Department of Pediatrics, University of Washington, Seattle, Washington USA; 6 Genome Sciences, University of Washington, Seattle, Washington USA; 7 Experimental Immunology, Allen Institute, Seattle, Washington USA

**Correction to: Pediatr Rheumatol 18, 80 (2020)**

**https://doi.org/10.1186/s12969-020-00474-1**

The original publication of this article [1] contained 2 errors in the nomenclature of the HMOX1 deletion. The incorrect and correct nomenclature is shown below. The original article has been updated.

**Incorrect**

**Abstract**

- HMOX1 c.264_269delCTGG (p.L89Sfs*24)

**Genetic analysis section**

- HMOX1 c.264_269delCTGG (p.L89Sfs*24)

**Correct**

**Abstract**

- HMOX1 (NM_002133.3):c.262_268delinsCC (p.Ala88Profs*51)

**Genetic analysis section**

- HMOX1 (NM_002133.3):c.262_268delinsCC (p.Ala88Profs*51)
