# Supplementary material for: Heme oxygenase-1 deficiency presenting with interstitial lung disease and hemophagocytic flares
Source: Pediatr Rheumatol Online J. 2020 Oct 16;18:80. doi: 10.1186/s12969-020-00474-1 (PMC7565350; doi:10.1186/s12969-020-00474-1)
Supplement: Supplementary file 1 — Additional file 1: Table S1. Summary of previously published HMOX1 deficiency cases. [file 12969_2020_474_MOESM1_ESM.docx]

**Supplemental Table 1. Summary of previously published *HMOX1* deficiency cases**

| **Human HMOX1 Deficiency Cases** | Yachie *et al.* *J Clin Invest* 1999;103:129–135.  Kawashima *et al*.*Hum Path* 2002;33(1):125-130. | Radhakrishnan *et al*.  *J Pediatr Hematol Oncol* 2011;33:74–8. | Radhakrishnan *et al*.  *Pediatr Hematol Oncol* 2011;28:723–6. | Greil *et al*.  *Haematologica*  2016 Nov;101(11):e436-e439. | Gupta *et al*.  *Scand J Rheumatol*  2016;45(2):165-6. | Tahghighi *et al.*  *Int J Mol Cell Med*  2019;8(4):300-6. |
| --- | --- | --- | --- | --- | --- | --- |
| ***HMOX1* Gene Variants** | cDNA analysis:  Paternal c.322_325delCA  Maternal c.23_144del | Homozygous c.132A>T, p.R44X | Homozygous c.132A>T, p.R44X | Homozygous c.416G>T; p.G139V | Homozygous c.132 A>T, p.R44X | Homozygous c.A610T, p.K204X |
| **Symptom onset** | 26 months | 15 years | 2 years | 3 months | 20 months | 17 months |
| **Sex** | M | F | M | M | M | F |
| **Family History** | Mother with 2 missed abortions | No significant | No significant | Parents and brother with borderline microcytosis | No significant | Mother with 1 missed abortion |
| **Clinical Features** |  |  |  |  |  |  |
| **Systemic** | Recurrent fever  Growth retardation | Persistent fever | Fever  Failure to thrive | Persistent fever | Persistent fever | Persistent fever |
| **Dysmorphism** | Frontal prominence, saddle nose | Frontal prominence | Frontal prominence |  |  |  |
| **CNS** | Developmental delay  Subdural hemorrhage | Seizures due to right parietal hemorrhage |  |  |  |  |
| **Cardiac** | Hypertension  Left ventricular hypertrophy |  |  |  |  | Massive pericardial effusion drained by chest tube |
| **Lung** | Edema  Focal alveolitis  Microthrombi | Diffuse alveolar hemorrhage |  |  |  | Flares with tachypnea and respiratory distress |
| **Kidney** | Iron deposits | Nephritis | Nephrotic syndrome |  |  |  |
| **GI** | Hepatomegaly with iron deposits | Hepatitis | Hepatomegaly | Hepatomegaly | Hepatomegaly | Hepatitis  Hepatomegaly; liver biopsy (iron deposits) |
| **MSK** | Arthalgia, normal x-ray |  |  |  |  |  |
| **Heme/Lymph** | Asplenia  Cervical LAD  Coagulopathy  Hemolytic anemia (DAT negative)  Low bilirubin  Thrombocytosis | Asplenia  Coagulopathy  Cold agglutinin positive  Hemolytic anemia (DAT positive)  LAD  Low bilirubin  Thrombocytosis | Asplenia  Coagulopathy  Hemolytic anemia (DAT positive)  LAD  Low bilirubin  Thrombocytosis | Coagulopathy  Decreased NK cell cytotoxicity  Elevated ferritin  Microcytic anemia  Splenomegaly | Hemolytic anemia (DAT negative)  Hyposplenia  Leukocytosis  Low bilirubin  Thrombocytosis | Coagulopathy  Hemolytic anemia (DAT negative)  Leukocytosis  Low bilirubin  Normal spleen size  Thrombocytosis |
| **Hemophagocytosis** |  |  | Hemophagocytosis (bone marrow) | Hemophagocytosis (bone marrow, liver) |  | Normal bone marrow |
| **Skin** | Generalized erythematous rash | Diffuse maculopapular rash |  |  |  |  |
| **Infections** |  | *Trichosporon asahii* sepsis | Candidemia |  |  | *Escherichia coli* UTI |
| **Laboratory Findings**  **(max reported)** | LDH 17,470 IU/L  Ferritin 780 ng/mL  D-dimer elevated | LDH 9,462 IU/L  Ferritin 4,912 ng/mL  AST 982 IU/L  D-dimer elevated | LDH 12,858 IU/L  D-dimer elevated  Ferritin 15,530 ng/ml | LDH 15,713 IU/L  Ferritin 11,044 ng/ml | Ferritin >2,000 ng/ml | LDH 15,350 IU/L  Ferritin 27,425 ng/mL  AST 580 IU/L  TG 723 mg/dL |
| **Treatment** |  | PRBC transfusions  Methylprednisolone 20 mg/kg/day  Prednisone 2 mg/kg/day  Cyclophosphamide  Rituximab | PRBC transfusions  Methylprednisolone | PRBC transfusions  Dexamethasone  Etoposide  Cyclosporine A  Phlebotomy |  | Methylprednisolone 30 mg/kg/day  Prednisone |
| **Outcome** | Death at 6-years-old from intracranial hemorrhage | Death 5 months after presentation from intracranial hemorrhage | Death 2 years old, transfusion dependent, fever and pallor. | Alive at 10-years-old | Death 3 months after diagnosis (23 months) | Death at 2 years from recurrent fever, hemorrhage, heart failure, and ascites |

AST – aspartate aminotransferase, CNS – central nervous system, DAT – direct antiglobulin test. GI – gastrointestinal, LAD – lymphadenopathy, LDH – lactate dehydrogenase, MSK – musculoskeletal, NK – natural killer, PRBC – packed red blood cell, TG – triglycerides, UTI – urinary tract infection
